# Interactions among multiple stressors vary with exposure duration and biological response

**DOI:** 10.1098/rspb.2022.0348

**Published:** 2022-05-11

**Authors:** Olivia C. King, Jason P. van de Merwe, Max D. Campbell, Rachael A. Smith, Michael St. J Warne, Christopher J. Brown

**Affiliations:** ^1^ Coastal and Marine Research Centre, Australian Rivers Institute, School of Environment and Science, Griffith University, Gold Coast, Queensland 4222, Australia; ^2^ Office of the Great Barrier Reef, Department of Environment and Science, Queensland Government, Brisbane, Queensland 4000, Australia; ^3^ School of Earth and Environmental Sciences, University of Queensland, Brisbane, Queensland 4067, Australia; ^4^ Water Quality and Investigations, Queensland Department of Environment and Science, Brisbane, Queensland 4102, Australia; ^5^ Centre for Agroecology, Water and Resilience, Coventry University, West Midlands, CV1 5FB, UK

**Keywords:** interaction, multiple stressors, coastal ecosystems, photosystem II herbicides, light availability, dissolved inorganic nitrogen

## Abstract

Coastal ecosystems are exposed to multiple anthropogenic stressors. Effective management actions would be better informed from generalized predictions of the individual, combined and interactive effects of multiple stressors; however, few generalities are shared across different meta-analyses. Using an experimental study, we present an approach for analysing regression-based designs with generalized additive models that allowed us to capture nonlinear effects of exposure duration and stressor intensity and access interactions among stressors. We tested the approach on a globally distributed marine diatom, using 72 h photosynthesis and growth assays to quantify the individual and combined effects of three common water quality stressors; photosystem II-inhibiting herbicide exposure, dissolved inorganic nitrogen (DIN) enrichment and reduced light (due to excess suspended sediment). Exposure to DIN and reduced light generally resulted in additivity, while exposure to diuron and reduced light resulted in additive, antagonistic or synergistic interactions, depending on the stressor intensity, exposure period and biological response. We thus find the context of experimental studies to be a primary driver of interactions. The experimental and modelling approaches used here bridge the gap between two-way designs and regression-based studies, which provides a way forward to identify generalities in multiple stressor interactions.

## 1. Background

Most of Earth's ecosystems are exposed to multiple stressors [1–3]. Biodiversity loss or the degradation of ecosystem services cannot be appropriately managed without approaches that address multiple, interacting stressors. Management of stressors is vastly improved when the impacts on ecosystems can be predicted or quantitatively measured [4]. However, in most cases, empirical data on multi-stressor interactions are lacking, meaning that combined or interacting effects are not often considered or assessed by management bodies [1,4–6]. Thus, management would benefit from general models that predict how stressors interact, based on theoretical deduction. A common approach to predicting stressor interactions is to seek generalities through meta-analyses that classify interactions as additive, antagonistic or synergistic [7]; or, through variations of this simple scheme [1,4,8]. Additive interactions mean that the combined effects of stressors are indistinguishable from the sum of their individual effects on an ecological response, whereas synergism and antagonisms describe outcomes where the combined effects are more or less than the additive response, respectively [4,7]. These classifications are useful, as each has different implications for conservation and management. The most recent reviews have shifted from focusing solely on synergies, which accelerate the impacts of multiple stressors on biodiversity [4,9], to also focusing on antagonisms [10,11] and other non-additive interactions [12], which may mitigate future environmental impacts.

A challenge with trying to infer generalities in interaction types is that meta-analyses are typically unable to capture important context about the intensity of the stressor or the timescale of the experiment [10,13,14]. When organisms respond nonlinearly to stressors, the interaction types that are analysed with classic additive models will appear to change with stressor intensity [15]. Further, the interactive effects of stressors have been shown to vary across levels of biological organization [4,10]. As such, few generalities in stressor interactions have emerged from meta-analyses, which limits our ability to predict interaction types [12]. Overall impacts of stressors on an organism may arise from physiological (cellular), whole-organism (organismal) and/or population (community) processes [10–12]. Data and theory support a greater occurrence of antagonisms at the community level, versus synergies and additive effects at physiological and population levels [4,13]. The result of additive, synergistic or antagonistic interactions at different levels of biological organization may vary depending on factors such as stressor magnitude, exposure duration and the specific endpoint measured [11,16]. For example, measurements of multiple endpoints are required to determine overall individual and interactive effects between two common stressors in coastal environments, herbicide pollution and light limitation [17]. For herbicide and reduced light exposure, multiple endpoints are required to determine overall individual and interactive effects. This is because direct measurements of photophysiological responses (i.e. chlorophyll-a fluorescence) will not account for biochemical responses such as intracellular damage, which then ultimately impact whole-organism responses (i.e. growth and mortality) as secondary sites of impact [11]. Therefore, there is a need for empirical studies that explore how interaction types change over multiple levels of biological organization and over time, and for experiments that use regression-type designs to study how stressor intensity affects interactions [12,14].

Quantifying how experimental context affects interaction types has been identified in recent reviews as a key priority for the field of multiple stressor research [12,14]. Here, we conducted an experimental study to quantify how interaction types vary when the globally distributed marine diatom, *Phaeodactylum tricornutum* was exposed to different intensities of stressors, over acute and chronic exposure periods. Two biological responses (photosynthetic and growth inhibition) were used to measure the effects of three common water quality stressors. These stressors include dissolved inorganic nitrogen (DIN) enrichment, diuron (a photosystem II (PSII)-inhibiting herbicide) exposure and light limitation, which were expected to have both positive and negative effects on algae growth. These laboratory experiments act as an environmentally relevant case-study, as degrading water quality from common anthropogenic inputs has been identified as a substantial risk to marine and coastal ecosystems, globally [3,18,19]. Pesticides, nutrients and decreased light (from increased turbidity) are three key stressors threatening coastal ecosystems [20] and are expected to have interactive effects on primary producers (e.g. marine microalgae and seagrass) that vary across different levels of biological organization [11]. A marine diatom was chosen for use in this study, as they are important primary producers that form the base of many food-webs and contribute to nutrient cycling in marine waters [21,22]. Ammonium (${\mathrm{NH}}_{4}{}^{+}$) is a source of inorganic nitrogen that is readily available for phytoplankton uptake and assimilation [23,24]. Increased turbidity due to elevated total suspended sediments alongside increased phytoplankton biomass as a result of eutrophication cause a decrease in the amount of photosynthetically active radiation available to aquatic photosynthetic organisms such as seagrasses, macroalgae and microalgae [25–27]. Diuron, a PSII-inhibiting herbicide, was chosen based on Queensland catchment and marine monitoring data adjacent to the Great Barrier Reef (GBR) [20,28–31] that indicates it is one of the most frequently detected pesticides year-round in waters entering the GBR [32,33].

The aims of this research were (i) to predict the nonlinear effects of diuron exposure, DIN enrichment and reduced light on two response variables, photosynthesis and growth; (ii) to identify how interaction types for the two response variables vary over different exposure durations; and (iii) to compare the interaction types for the two response variables at the same experimental treatment levels. To address these aims, we used an experimental study and applied a novel approach to access stressor interaction types. This was done by integrating a regression-based block design with continuous nonlinear modelling, which subsequently translates the nonlinear effects into a standard interaction-type classification. This allowed us to assess how interactions change with experimental context. The innovation of this study is in the integration of the experimental design with the statistical framework for nonlinear analysis.

## 2. Methods

### (a) Multi-stressor laboratory-based exposure assays

Marine microalga, *P. tricornutum,* is a globally distributed primary producer in estuarine and coastal areas [34] and is considered to be an excellent model species for assessing physiological and biological endpoints in laboratory experiments [35]. *P. tricornutum* was exposed to diuron (C_9_H_10_Cl_2_N_2_O; analytical standard, greater than 95%) with reduced light availability; and DIN (NH_4_Cl; analytical standard greater than or equal to 99.5%) with reduced light availability as detailed in the electronic supplementary material, S1. Range finding tests were conducted to identify levels of each stressor that were likely to cause a 0–90% effect on a sensitive endpoint [36]. Definitive toxicity tests used the level at which an approximately 50% effect occurred as the highest exposure threshold (to allow for increased effect when combined with the other stressor). All toxicity tests were performed on four separate occasions (blocks), using independent algae cultures. Paired combinations of environmentally relevant levels of diuron, DIN and light reduction were tested under laboratory conditions. For the DIN experiments, a total of nine flasks were used per block, containing two NH_4_Cl treatments (2.76 and 27.6 mg l^−1^) plus an algae control, each at three light levels of 5, 20 and 80 µmol photons m^−2^ s^−1^ (*n* = 36). For the diuron experiments, 18 flasks were used per block, containing four diuron concentrations (0.1, 0.3, 1 and 3 µg l^−1^) plus a methanol control (at 0.08%) and an algae control, each at three light levels of 5, 20 and 80 µmol photons m^−2^ s^−1^ (*n* = 72).

Measurements of photosynthetic yield, determined as effective quantum yield of PSII measured using variable chlorophyll-a fluorescence [37], were recorded at 0 (immediately before), 0.3 (immediately after), 2, 24, 48 and 72 h after stressor addition. Measurements of cell density (growth), determined via measures of spectrophotometric absorbance at 685 nm (OD_685_) [34], were recorded at 0, 48 and 72 h after stressor addition. A regression-based experimental design was used to enable the study of how interactions varied at different stressor intensities [14,38].

### (b) Identifying interaction types

The framework used to classify interactions is critical [4,39,40], because it influences the prevalence of different interaction types. Here, we developed a nonlinear continuous modelling framework that (i) enabled us to predict how interactions varied across time and stressor intensities and (ii) predicted uncertainty intervals under these different contexts. Gaussian generalized additive models (GAMs) with identity links were used to model the interactive effects of multiple stressors (diuron, DIN and reduced light) on *P. tricornutum*, for the two biological response variables across all samples and exposure durations.

The measured biological responses (*R_t,i,j_*) for the growth and photosynthesis models were cell density and effective quantum yield, respectively. Cell density values were ln-transformed for use in the models because algal cells grow exponentially, and thus, the residuals were heteroscedastic on the untransformed scale. Models were formulated as per equation (2.1).

2.1 $$R_{t,i,j}=c_{i,j}+f_{1}(t)+f_{2}(L_{i},S_{i},t)+g_{1}(j)+{\;}_{}\epsilon_{t,i,j,}$$

where *R_t,i,j_* is the measured biological response at time *t* in sample *i,* and block *j* (equation (2.1)), *c* is an offset that accounted for the starting values of the response (i.e. photosynthetic yield and ln-transformed cell density, at time zero), for each sample (*i*), *f*_1_(*t*) is a one-dimensional thin-plate smoother for time, *f*_2_ is a two-dimensional thin-plate smoother that captured the interaction between the level of light (*L_i_*) in the sample and the level of the other stressor (*S_i_*; either diuron or DIN) at each time point (*t*); *g* is the penalized random effect term to account for natural differences among blocks (*j*) and *ɛ* is an error term [41].

Visual checks of the model residuals were undertaken to ensure that the model was appropriately specified, and that the assumptions for homoscedasticity, normality and independence were not violated. These checks revealed all model assumptions to be verified. The models were fitted with the R statistical software package, ‘mgcv’ [41].

The mean of the measured biological responses at each time point (*R_t_*) was predicted with 95% credible intervals computed using the empirical Bayesian approach, following the resampling procedure in [41]. We interpreted effect sizes by reference to the 95% credible intervals. The 95% credible intervals are conservative in that they marginalize over all variation in random effects, so they represent effect sizes relative to all modelled sources of variation.

We then predicted the interactive effects from the fitted GAMs. Interaction effects were measured with a statistic that represented the type of interactions that would be observed in a two-way experimental design (*I_R_*); equation (2.2)). The *I_R_* statistic was predicted with 95% credible intervals as above. Control treatments were set at 0 µg l^−1^ of diuron, 0 mg l^−1^ of DIN and 80 µmol photons m^−2^ s^−1^ of light. The simple statistic (*I_R_*) incorporates the predicted mean value of the biological response at a time *t* variable. We denoted the predicted response values (*Y*) as *Y_t_*_A_, *Y_t_*_B_, *Y_t_*_AB_ and *Y_t_*_C_, where the terms A, AB and C refer to the treatments with either stressor A, B, both A and B or the control, respectively. The *I_R_* statistic produced a negative value for a synergistic interaction, because the denominator of the fraction is larger than the numerator (i.e. ln(*x*) less than 0, when 0 < *x* <1). Similarly, the *I_R_* statistic produced a positive value for an antagonistic interaction and zero for no (additive) interaction.

2.2 $$\begin{array}{rl} I_{R} & =(\ln(Y_{t\mathrm{AB}})-\ln(Y_{tC}))-(\left(\ln(Y_{tA})-\ln(Y_{tC}\right))\\ & \;+\;(\ln(Y_{tB})-\ln(Y_{tC})))\\ & =\ln\left(\frac{Y_{t\mathrm{AB}}/Y_{tC}}{Y_{tA}Y_{tB}/Y_{tC}^{2}}\right). \end{array}$$

This approach compares the observed response to the combined stressors (after accounting for the block and the starting concentration) to the response predicted by the independent action (IA) model of joint action. The IA model is commonly used in ecotoxicology analyses to quantify the environmental impact of multiple stressors on organisms [42]. Using our approach, an *I*_*R*_ = 0 characterizes an observed interaction that is the same as the IA model. This approach was then tested with simulated data to verify whether it was able to accurately identify interaction types in idealized scenarios (electronic supplementary material, S2).

## 3. Results

### (a) Individual and paired responses

**Figure 1.**
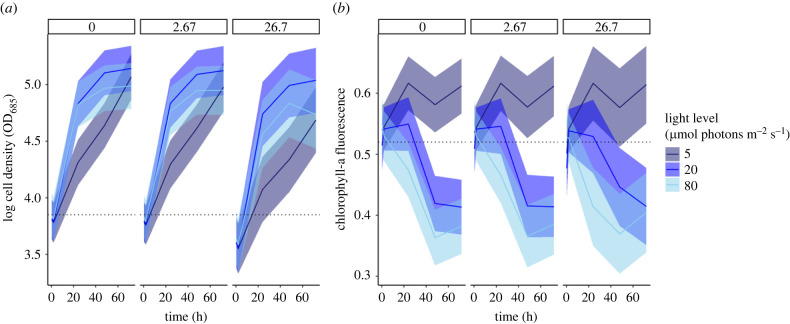
The predicted mean response (solid lines) with 95% confidence intervals (shading) of (*a*) cell density and (*b*) chlorophyll-a fluorescence following exposure to DIN (individual panels; mg l^−1^) and reduced light (colours; µmol photons m^−2^ s^−1^) over time. Dotted horizontal lines indicate the starting cell density in (*a*) and chlorophyll-a fluorescence units in (*b*), at hours = 0 (immediately before stressor addition). The first measurements begin at hours = 0.33 (i.e. approximately 20 min, or immediately after stressor addition). (Online version in colour.)

Cell density (growth) of the lowest DIN treatment (2.76 mg l^−1^) under all light scenarios was similar to the controls (95% confidence intervals overlapped the means) (figure 1*a*). Visible reductions of cell density occurred in the highest DIN treatment (26.7 mg l^−1^); however, a marked reduction of cell density was only observed when high DIN was coupled with the lowest light treatment (5 µmol photons m^−2^ s^−1^; figure 1*a*).

Chlorophyll-a fluorescence (photosynthetic efficiency) of both DIN treatments was similar to the controls (95% confidence intervals overlapped the means) (figure 1*b*). When exposed to increased DIN and reduced light in combination, photosynthetic efficiency remained high, while cell density (growth) was reduced (figure 1*a,b*).

**Figure 2.**
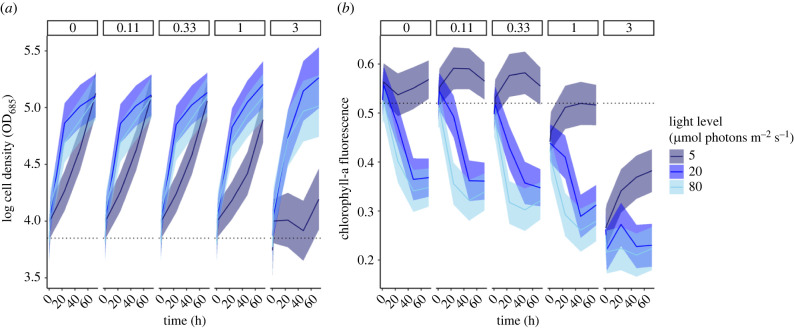
The predicted mean response (solid lines) with 95% confidence intervals (shading) of (*a*) cell density and (*b*) chlorophyll-a fluorescence following exposure to diuron (individual panels; µg l^−1^) and reduced light (colours; µmol photons m^−2^ s^−1^) over time. Dashed horizontal lines indicate the starting cell density in (*a*) and chlorophyll-a fluorescence units in (*a*), at hours = 0 (immediately before stressor addition). The first measurements begin at hours = 0.33 (i.e. approximately 20 minutes, or immediately after stressor addition). (Online version in colour.)

As the intensity of diuron and shading increased, cell density decreased (figure 2*a*), with the largest decrease occurring when exposed to 3 µg l^−1^ diuron and the lowest light level of 5 µmol photons m^−2^ s^−1^. Chlorophyll-a fluorescence was affected by increasing diuron concentrations (figure 2*b*), with the largest decrease in chlorophyll-fluorescence occurring at 3 µg l^−1^ diuron and the highest light level of 80 µmol photons m^−2^ s^−1^, compared to the controls. Chlorophyll-a fluorescence was considerably reduced as early as 2 h following exposure to high diuron (3 µgl^−1^) treatments under all light scenarios (figure 2*b*).

Photosynthetic efficiency (chlorophyll-a fluorescence) was a more sensitive endpoint than cell density (growth) when measuring the individual and paired effects of diuron exposure, DIN enrichment and reduced light on *P. tricornutum*. Generally, the predicted responses were negatively affected when *P. tricornutum* was exposed to DIN or diuron under 5 µmol photons m^−2^ s^−1^, indicating interactions among stressors. For example, high exposure to diuron had little effect on cell density (growth) at the higher light levels but the effect was substantially reduced at the lowest light level (figure 2*b*).

### (b) Interaction responses

**Figure 3.**
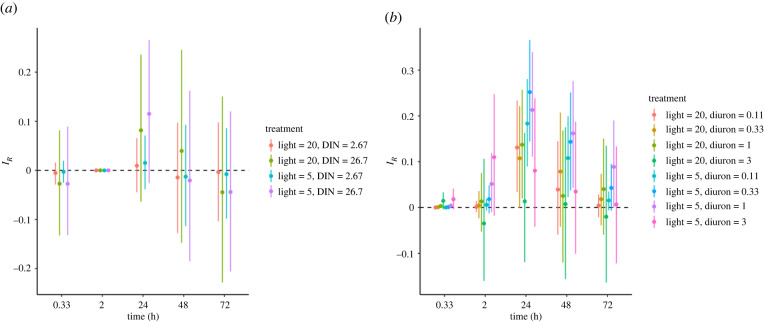
The change in median interactive effects (*I_R_*) from multiple stressors; (*a*) DIN (mg l^−1^) and reduced light (µmol photons m^−2^ s^−1^) and (*b*) diuron (µg l^−1^) and reduced light (µmol photons m^−2^ s^−1^) over time for chlorophyll-a fluorescence. Each plot displays the median estimated effect (*I_R_*; points) with 95% credible intervals computed using the empirical Bayesian approach. Positive and negative *I_R_* values indicate antagonistic and synergistic interactions between stressors, respectively. The black dashed lines show *I_R_* = 0, which represents no stressor interaction or an additive interaction where their combined effects are the sum of the individual treatment effects.

There was limited evidence for an interactive effect of DIN and reduced light on chlorophyll-a fluorescence (figure 3*a*), as the credible intervals for *I_R_* overlapped 0, and the interaction between diuron and reduced light was generally additive (i.e. approximately 80% of treatments) on chlorophyll-a fluorescence (figure 3*b*).

Antagonisms occurred for some diuron and low light combinations at 24 and 48 h (figure 3*b*). Notably, all treatment combinations except those with the highest diuron concentration had antagonisms at 24 h. The strength of the antagonisms weakened between 24 and 48 h, with some combinations shifting to being indistinguishable from additivity. By 72 h, antagonisms were only marginally significant for the three lowest diuron concentrations (0.11 to 1 µg l^−1^) at the lowest light level (figure 3*b*).

It is unlikely that the photosynthetic effects of light would be triggered at 0.33 and 2 h, resulting in an antagonistic interaction or no interactive effect; thus, the outcomes at these time points should be treated with caution (figure 3*a,b*). For example, when high diuron (3 µg l^−1^) was coupled with low light at 0.33 and 2 h, there was a high probability (greater than 0.99) of antagonistic effects on photosynthetic inhibition (figure 3*b*) which can be explained by the lack of effect from light. When the effects of light on photosynthetic inhibition begin to occur at 24 h exposure, all combinations of diuron and reduced light were highly antagonistic, except for three treatment combinations where the 95% credible intervals overlapped with the *I_R_* equals zero line. The highest diuron concentration (3 µg l^−1^) coupled with 20 and 5 µmol photons m^−2^ s^−1^ light levels had median estimated effect values that suggested antagonisms but with 95% credible intervals that also suggested strong potential for additivity or synergy (figure 3*b*). In addition, the 0.33 µg l^−1^ diuron treatment coupled with 20 µmol photons m^−2^ s^−1^ light had credible intervals that overlapped *I_R_* = 0, meaning the effect could not be statistically differentiated from an additive interaction (figure 3*b*).

**Figure 4.**
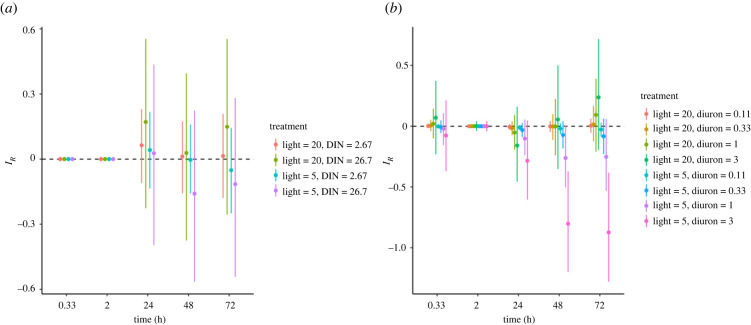
The change in median interactive effects (*I_R_*) from multiple stressors; (*a*) DIN (mg l^−1^) and reduced light (µmol photons m^−2^ s^−1^) and (*b*) diuron (µg l^−1^) and reduced light (µmol photons m^−2^ s^−1^) over time for growth inhibition. Each plot displays the median estimated effect (*I_R_*; points) with 95% credible intervals computed using the empirical Bayesian approach. Positive and negative *I_R_* values indicate antagonistic and synergistic interactions between stressors, respectively. The black dashed lines show *I_R_* = 0, which represents no stressor interaction or an additive interaction where their combined effects are the sum of the individual treatment effects.

There was also limited evidence for an interactive effect of DIN and reduced light on growth inhibition (figure 4*a*), thus the interaction could not be distinguished from an additive null for all treatment combinations over the 72 h exposure period. The lack of interactive effects could be explained by the limited effects of DIN at the intensities tested.

Diuron and reduced light likely had no interactive effect on growth inhibition at 0.33 and 2 h (figure 4*b*). At 24 h, synergistic responses for growth inhibition became stronger (higher probability) as diuron concentrations increased, under reduced light and over time (figure 4*b*). At 20 µmol photons m^−2^ s^−1^ of light and 3 µg l^−1^ of diuron, growth inhibition was likely to be synergistic at 24 h; however, the probability of additivity or antagonisms increased at 48 and 72 h (figure 4*b*). At 5 µmol photons m^−2^ s^−1^ light, the high diuron treatments (1 and 3 µg l^−1^) were strongly synergistic for growth inhibition at 48 and 72 h (figure 4*b*).

Simulation testing of the nonlinear modelling was undertaken for both endpoints (i.e. photosynthetic and growth efficiency) by classifying known stressor interactions (electronic supplementary material, S2). It was found that the model correctly detected and classified stressor interaction types with accuracy. The model performed consistently better than using a raw statistic for both endpoints when the natural variation was low to moderate, and the stressor interactions were stronger.

## 4. Discussion

The predicted nonlinear effects of diuron, nutrient (DIN) enrichment and reduced light on *P. tricornutum* changed depending on the duration of the experiment and the intensity of the stressors. Our approach of applying GAMs to translate these nonlinear effects into standard interaction types revealed that non-additive interaction types were more prevalent in the diuron and reduced light treatments, compared to the DIN and reduced light treatments. Generally, DIN and reduced light resulted in additive interactions, while diuron and reduced light resulted in additive, antagonistic or synergistic interactions, depending on the stressor intensity, exposure period and the measured biological response.

Each individual stressor exerts its impact via a specific mode of action, meaning that the type of interaction observed is dependent on multiple direct and indirect pathways of impact [43,44]. It is important to understand the physiology of the study organism's response to stressors, as this drives the nonlinearities in organismal response that underpin general patterns with interactions. The interactive effects observed in this study are consistent with the physiological responses underlying the algal stress-response, suggesting that this knowledge of the mode of action will help predict interactive effects in unmeasured contexts.

Photosynthetic activity is a specific and sensitive bioindicator of plant stress and is quick and easy to measure [45–47]. Photosynthetic efficiency is strongly affected by diuron and other PSII-inhibiting herbicides, as these herbicides block electron transport in the PSII-complex, inhibiting photosynthesis [48]. Thus, interactive effects for photosynthetic inhibition were observed much sooner at 2 h or less, compared to that of growth inhibition observed at 24 h or more. This is because diuron can inhibit photosynthesis almost immediately, indicating a specific effect on the algae due to the direct impairment of PSII [49], while later readings may indicate delayed phytotoxicity and include both specific and non-specific effects [50]. By contrast, reduced light can decrease (rather than block) electron transport [51] but increase effective and maximum quantum yields of PSII—ultimately leading to increased photosynthetic efficiency [52,53]. Additionally, pigment content (e.g. chlorophyll) can increase to compensate for low-light stress, increasing photon capture that also increases photosynthetic efficiency [54]. However, high photosynthetic efficiency or pigment does not necessarily correlate to increased growth.

As exposure to diuron decreases photosynthetic efficiency, reduced light activates compensatory responses within the plant that increase photosynthetic efficiency. This compensation may explain the antagonistic interactions that were observed between the two stressors at 24 to 48 h. Antagonisms were also predicted when assessing the combined effects of diuron and low-light stress on photophysiological responses (i.e. photosynthetic efficiency) and biochemical responses (i.e. oxidative stress and energetic reserves) [11], which typically occur within hours to days following exposure [17]. As exposure to low-light stress gets longer (24 h or more), compensatory photosynthetic responses become exhausted, thus decreasing photosynthetic efficiency [55]. As a result, our observations were consistent with previous hypotheses that the interactive effect of diuron and low-light stress would be antagonistic at shorter time-scales (i.e. less than 48 h), but switch to additive or synergistic interactions after longer exposure durations (i.e. greater than 24 h) [11].

The strong synergistic effects on growth when algae were exposed to high diuron concentrations and the low-light levels were consistent with earlier hypotheses of synergistic interactions as the stressor magnitudes intensify [11], and are consistent with literature findings that reported a lower toxic response when marine microalgae were exposed to a mixture of PSII-inhibiting herbicides in lower light conditions, compared to higher light conditions [56]. Growth inhibition of microalgae is influenced by multiple underlying physiological responses. Diuron and/or low-light stress both impact photophysiology (photosynthetic efficiency), as found in this study, but also biochemistry (pigment content, reactive oxygen species (ROS) and oxidative stress) and whole-plant (growth and mortality) responses [17]. Diuron inhibits photosynthesis and increases the formation of ROS [48,57], where chronic low-light stress may maintain or reduce ROS concentrations via increased ROS-scavenging enzymes [58–60]. Prolonged exposure to elevated concentrations of ROS can cause irreversible cell damage, inhibit growth and lead to cell death [57]. As the effects of low-light stress generally take longer to appear (more than 24 h) and cytotoxic responses (i.e. cell damage) occur after approximately 24 h of exposure, all compensatory processes mentioned earlier would likely be exhausted. Therefore, the synergy we observed at longer exposure durations and higher stressor intensities may be explained by multiple physiological pathways for diuron and low-light stress, which negatively affect growth.

Multi-stressor studies are simplifications of complex systems and therefore have several limitations. First, meta-analyses from laboratory-based experiments should be interpreted with caution, as the extrapolation of laboratory conditions to field scenarios is challenging [8]. For example, the frequency and intensity of co-occurring stressors vary both spatially and temporally; however, laboratory experiments do not yet capture that degree of complexity [14]. Second, multi-stressor experiments are laborious and costly, often leading to low levels of replication that can hinder the detection of true stressor interactions [61]. Future studies would benefit from measuring additional biological, physical and/or chemical response variables such as ROS and photosynthetic pigments or by-products of chemical reactions, to further understand the interactive effects of PSII-inhibiting herbicides, reduced light and increased DIN. Finally, increasing the size of the experimental dataset may decrease parameter uncertainty and credible intervals within the modelling component of this study. This means that replicating experiments would help strengthen generalities in nonlinear effects [61].

## 5. Conclusion

Previous studies have called for regression-based experimental designs to predict interaction types among multiple stressors [12,14], because interaction types will vary with experimental context such as stressor intensity and the exposure duration at which responses are measured [12,16,62]. This study showed how GAMs can be applied to translate nonlinear stressor effects into a common interaction classification, to illustrate the importance of experimental context when determining interaction types. Interaction types were not consistent across the different experimental treatments, and notably could change for different response variables measured at the same time. These changes were explained by the physiological processes underlying the algae stressor responses. These results emphasize why meta-analyses of interaction types have largely failed to find generalities across studies that used simple two-way designs, because interactions are an emergent outcome of organism responses that change depending on stressor intensity and exposure duration. Thus, we concur with calls for future experimental studies to use regression-based designs [12,14] and nonlinear modelling [15]. With sufficient studies, meta-analyses should classify interactions based on contextual variables and the underlying stressor/s mode of action. This approach will help advance predictive models for stressor responses that are required for the management of important ecosystems facing multiple stressors, such as the GBR.

## Data Availability

Scripts and data to perform the analyses can be found at Dryad Digital Repository: https://doi.org/10.5061/dryad.6m905qg22 [63]. The data are provided in the electronic supplementary material [64].
